# High prevalence and diversity of extended-spectrum β-lactamase and emergence of OXA-48 producing *Enterobacterales* in wildlife in Catalonia

**DOI:** 10.1371/journal.pone.0210686

**Published:** 2019-08-05

**Authors:** Laila Darwich, Anna Vidal, Chiara Seminati, Andreu Albamonte, Alba Casado, Ferrán López, Rafael A. Molina-López, Lourdes Migura-Garcia

**Affiliations:** 1 Departament de Sanitat i Anatomia Animal, Universitat Autònoma de Barcelona (UAB), Cerdanyola del Vallès, Spain; 2 IRTA, Centre de Recerca en Sanitat Animal (CReSA, IRTA-UAB), Campus de la Universitat Autònoma de Barcelona, Cerdanyola del Vallès, Spain; 3 Catalan Wildlife Service, Centre de Fauna Salvatge de Torreferrussa, Santa Perpètua de Mogoda, Barcelona, Spain; University of Georgia, UNITED STATES

## Abstract

Most of the studies focused on antimicrobial resistance (AMR) performed in wildlife describe *Escherichia coli* as the principal indicator of the selective pressure. In the present study, several species of *Enterobacterales* with a large panel of cephalosporin resistant (CR) genes have been isolated from wildlife in Catalonia. A total of 307 wild animals were examined to determine the prevalence of CR enterobacteria, AMR phenotypes and the presence of common carbapenem and CR genes. The overall prevalence of CR-phenotype was 13% (40/307): 17.3% in wild mammals (18/104) and 11.5% in wild birds (22/191) (p<0.01). Hedgehogs showed the highest prevalence (13.5% of 104) of the mammal specimens, and raptors the highest in bird specimen (7.3% of 191). Although CR *E*. *coli* was the most frequently isolated (45%), other CR- *Enterobacterales* like *Klebsiella pneumoniae* (20%), *Citrobacter freundii* (15%), *Enterobacter cloacae* (5%), *Proteus mirabilis* (5%), *Providencia* spp (5%) and *Serratia marcescens* (2.5%) were also isolated. A high diversity of CR genes was identified among the isolates, with 50% yielding *bla*CMY-2, 23% *bla*SHV-12, 20% *bla*CMY-1 and 18% *bla*CTX-M-15. Additionally, resistance to carbapenems associated to OXA-48 gene was found. Most of the CR isolates, principally *K*. *pneumoniae* and *C*. *freundii*, were multi-resistant with co-resistance to fluoroquinolones, tetracycline, sulphonamides and aminoglycosides. This study reports high prevalence of *Enterobacterales* harbouring a variety of CR genes and OXA-48 mediated-carbapenem resistance, all of them frequently associated to nosocomial human infections, for the first time in wild mammals and wild birds. Implementation of control measures to reduce the impact of anthropogenic pressure in the environment is urgently needed.

## Introduction

In the last decades, the prevalence of opportunistic and antimicrobial resistant (AMR) bacteria associated with nosocomial infections has increased in hospital settings. The overuse of antibiotics in human and veterinary medicine have led to the spread of AMR pathogens, becoming a global health problem [1].

Extended-spectrum β-lactamases (ESBLs) and AmpC-type β-lactamases (AmpC) are the most common enzymes that confer resistance to broad-spectrum cephalosporins among members of the family *Enterobacterales*. These β-lactamases have extensively diversified in response to the clinical use of new generation drugs: cephalosporins, carbapenems and monobactams [2]. There are currently two classification systems for beta-lactamase enzymes. The first one classifies beta-lactamases according to the amino acid sequence [3,4]. The second classification, described by Bush and Jacoby (2010) is based on the functional activity of the enzymes. Within this classification, the group 1 contains cephalosporinases encoded in the chromosome of many *Enterobacterales*, such as *AmpC*, CMY, ACT, FOX and MIR. Some variants of these enzymes have also been detected in plasmids. The group 2 serine beta-lactamase represents the largest group with a broad spectrum against penicillins, cephalosporins, and carbapenems. They include the TEM, SHV, CTX, OXA and KPC enzymes. These enzymes are mostly encoded by genes located in plasmids that can be horizontally transferred to different bacteria genera [1]. Finally, the group 3 metallo-beta-lactamases (MBLs) are zinc dependent and include NDM, IMP, VIM and SPM enzymes [5].

Carbapenems are last-line beta-lactam antibiotics with the broadest spectrum of activity. Nowadays, carbapenems are commonly used in hospital settings for the treatment of life-threatening infections caused by *Enterobacterales* resistant to beta-lactamic drugs, including cephalosporins, monobactams and inhibitors of beta-lactamases. However, the emergence of resistance to carbapenems mediated by the production of carbapenemases has led to limited therapeutic options in human health [6]. The OXA-48 variant of carbapenemases is becoming highly prevalent in human clinical infections [7].

The dissemination of cephalosporin resistance (CR) has been studied widely in *Enterobacterales* from humans and livestock, whereas studies concerning the environment, including wildlife, are still lacking [2]. In recent years, an important increase of CR *Escherichia coli* has been reported in different epidemiological settings such as humans, pets, livestock, retail meat and the environment [8–13]. The study of wildlife as sentinel of the AMR environmental contamination has recently acquired more consideration worldwide [14]. However, most of the environmental-wildlife interface studies have been focused on wild birds, as principal AMR disseminators by their migratory routes, with a limited variety of AMR bacteria species described. Isolation of CR-carrying bacteria from wild birds has been globally reported in *E*. *coli* [15–20] and less frequently in *Klebsiella pneumoniae* [21]. All these results confirm the dissemination success of ESBL *bla*_SHV-12_ and *bla*_CTX-M_ variants in wild birds worldwide. More recently, presence of CR *E*. *coli* has also been described in wild mammals, but at lower prevalence in comparison with wild birds [22].

In the present study, we report for the first time in Spain, the presence of diverse families of CR-encoding genes in a large variety of *Enterobacterales* including *E*. *coli*, *K*. *pneumoniae*, *Citrobacter freundii*, *Enterobacter cloacae*, *Serratia marcescens and Proteus mirabilis*- in wild mammals and wild birds. Furthermore, we describe the presence of carbapemenase resistant *E*. *coli* and *P*. *mirabilis* associated with the presence of OXA-48 variant in isolates of wildlife origin. These bacteria are frequently found in recurrent and severe urinary tract infections and other nosocomial infections in hospitals of Spain [7,23].

## Material and methods

### Study population

Wild animals attended at the Wildlife Rehabilitation Centre (WRC) of Torreferrusa (Catalonia, North-East Iberian Peninsula) were analysed between November 2016 and May 2017. This is a public WRC under the direction of the Catalan Wildlife-Service (“Direcció General de Polítiques Ambientals, Departament de Territori i Sostenibilitat of the Generalitat de Catalunya”). Sampling methods and handling protocols of animals were in agreement with the Catalan Wildlife Service who stipulates the management protocols and Ethical Principles according to the Spanish legislation [24]. All animals were examined and tested using cloacal or rectal swabs on arrival at the centre before receiving any pharmacologic or antimicrobial treatment. The most frequent cause of hospitalization was related to anthropogenic origin due to direct persecution (gunshot, poisoning, illegal captivity or traps) to involuntary human induced threats (collisions with vehicles, fences or electric lines and electrocution).

### Microbiological analysis

Rectal and cloacal swabs were plated in MacConkey agar supplemented with ceftriaxone (1mg/L). Single colonies growing on the plate were subculture and identified biochemically using API (bioMérieux, Marcy l’Etoile, France) or VITEK 2 (bioMérieux, Spain) systems.

### Antimicrobial susceptibility testing

Minimal inhibitory concentration (MIC) was performed using a commercial broth microdilution method (VetMIC GN-mo, SVA, Sweden) for the following antimicrobials: ampicillin (1 to 128 mg/liter), cefotaxime (0.016 to 2 mg/liter), ceftazidime (0.25 to 16 mg/liter), nalidixic acid (1 to 128 mg/liter), ciprofloxacin (0.008 to 1 mg/liter), gentamicin (0.12 to 16 mg/liter), streptomycin (2 to 256 mg/liter), kanamycin (8 to 16 mg/liter), chloramphenicol (2 to 64 mg/liter), florfenicol (4 to 32 mg/liter), trimethoprim (1 to 128 mg/liter), sulfamethoxazole (8 to 1,024 mg/liter), tetracycline (1 to 128 mg/liter), and colistin (0.5 to 4 mg/liter). The *E*. *coli* ATCC 25922 was used as control strain. Epidemiological cut-off values (ECOFF) selected were those described by the European Committee on Antimicrobial Susceptibility testing (EUCAST, https://mic.eucast.org/Eucast2/). For the combinations of species-antimicrobial with no cut-off values defined by EUCAST, ECOFF values were obtained from the British Society for Antimicrobial Chemotherapy (BSAC) or the Clinical and Laboratory Standards Institute (CLSI, 2017).

### Molecular characterization of antimicrobial resistance genes

The detection of genes coding for ESBLs -*bla*_CTX-M_ [25], *bla*_TEM_ [26], *bla*_SHV_ [27]-, AmpCs -*bla*_CMY-1_ [28], *bla*_CMY-2_ [29], carbapenemases -*bla*_OXA-48,_ *bla*_VIM,_ *bla*_IMP,_ *bla*_NDM_ and *bla*_KPC_ [30]- and colistin-resistance genes *mcr1-5* variants [31] was carried out using PCR as previously described (**S1 Table**).

Sanger DNA sequencing was done for *bla*_TEM_, *bla*_SHV_, *bla*_CTX-M_, and *bla*_OXA_ PCR products at the Genomic and Bioinformatics Service of the Universitat Autònoma de Barcelona (Spain). Sequences and chromatograms were manually explored to trim bad-quality bases with BioEdit 7.2. Once the assembly of the consensus sequences was done, partial sequences were aligned using Clustal Omega program, and finally blasted against the public database (National Center for Biotechnology Information, NCBI). Allelic variants of the ESBL-resistance genes were determined based on these partial sequences, and AmpC genes were classified according to the CMY-1 and CMY-2 groups.

#### Statistical analysis

Descriptive analysis was performed under 95% confidence, using SPSS Advanced Models TM 15.0 (SPSS Inc. 233 South Wacker Drive, 11th Floor Chicago, IL 60.606–6412). The Chi-square test or Fisher exact test was used for comparison between proportions when appropriate. Statistically significant results were considered for unadjusted p-value < 0.05.

## Results

**Fig 1:**
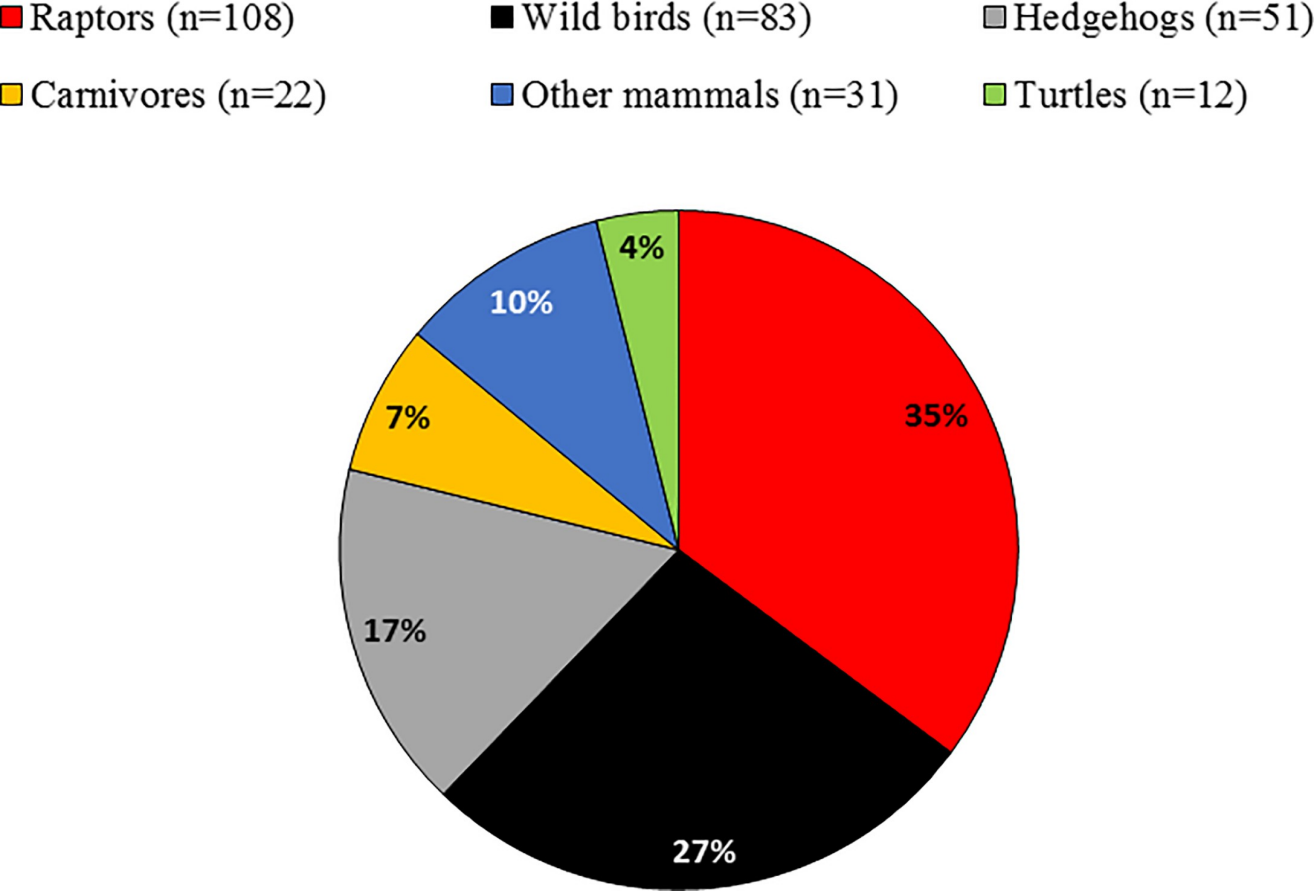
Proportion of wildlife analysed in the study according to the zoological category. Animal groups: raptors (different species of birds of prey and owls), wild birds (principally passerines and seagulls), insectivorous (European and Algerian hedgehogs), carnivores (mainly mustelids), and other mammals (wild boars and roe deer).

The sample size comprised 307 wild animals belonging to 67 different species grouped as birds (62%), mammals (34%) and reptiles (4%) (Fig 1). Animals came from different regions of Catalonia with a high density of urban areas and pig farming production.

**Fig 2:**
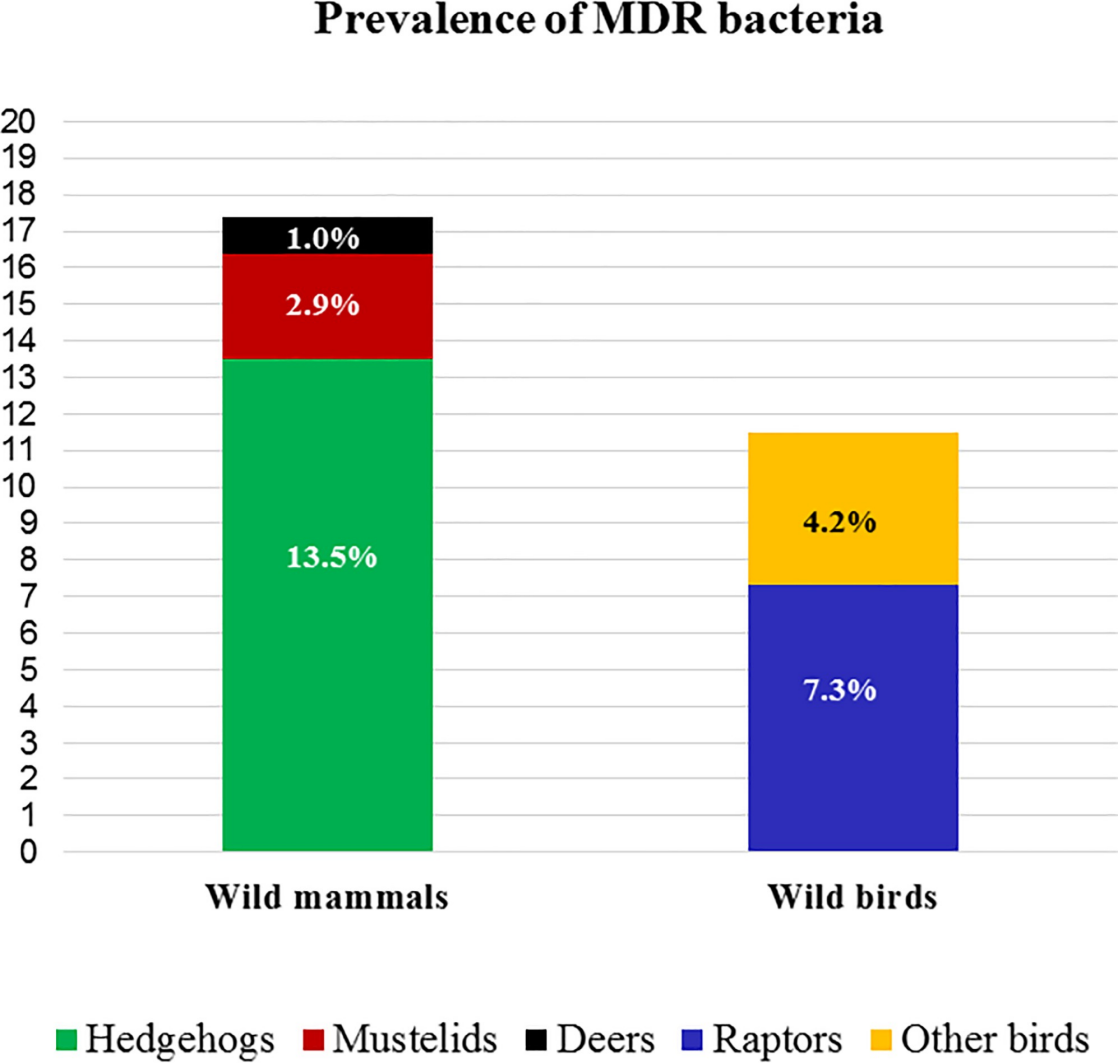
Prevalence of cephalosporin resistant (CR) bacteria in the different wildlife categories.

Ceftriaxone resistant isolates were detected in 65 out of the 307 (21%) faecal samples analysed. Of those, 40 harboured ESBL or AmpC-encoding genes, representing an overall prevalence of 13% (Fig 2). The prevalence of CR-carrying isolates was 17.3% in wild mammals (18/104) and 11.5% in wild birds (22/191). Within the mammal group, hedgehogs showed the largest prevalence of resistant isolates in comparison to the total mammal species examined (13.5%, 14/104, p = 0.022). Precisely, 67% of the Algerian (2/3) and 26% of the European (12/47) samples harboured CR-genes. Within the bird group, raptors represented the highest prevalence with 7.3% (14/191) of the total bird specimens [23% (14/60) of the raptor species examined] (Fig 2).

**Fig 3:**
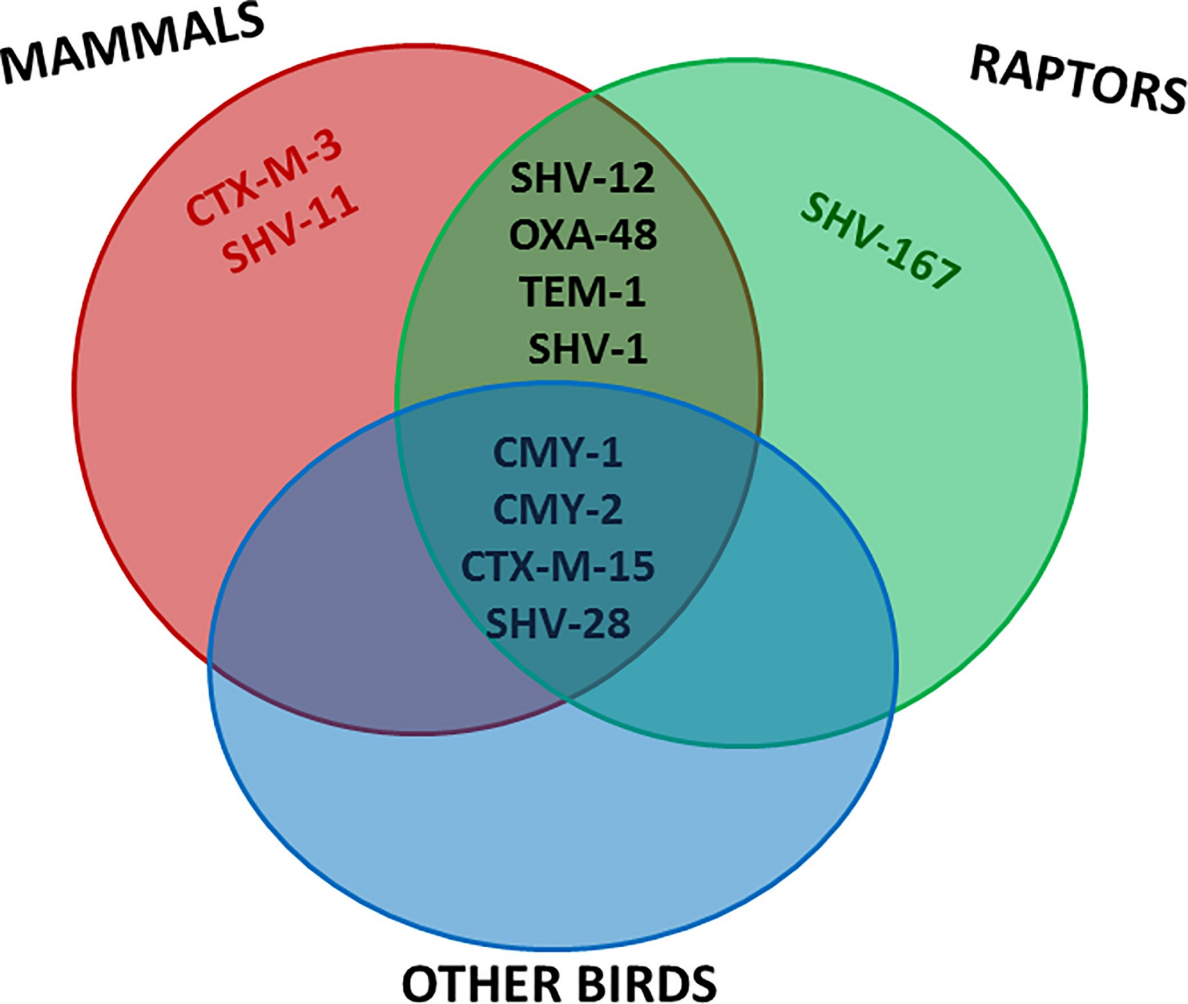
Venn diagram showing the distribution of AMR genes in the different animal groups encountered in this study.

CR isolates belonged to several genera within the Enterobacterales order, with *E*. *coli* being detected most frequently (45%). Interestingly, other clinically relevant enterobacteria, including *K*. *pneumoniae* (20%), *C*. *freundii* (15%), *E*. *cloacae* (5%), *P*. *mirabilis* (5%), *Providencia* spp (5%) and *S*. *marcescens* (2.5%) were also identified as carriers of CR genes. The proportion of positive samples for AmpC-encoding genes was 65% (26/40) distributed in 27% *bla*_CMY-1_ and 38% *bla*_CMY-2_ families. Additionally, 65% (26/40) of the isolates exhibited ESBL genes with *bla*_SHV-12_ (9/40, 22.5%) and *bla*_CTX-M-15_ (6/40, 15%) representing 35% and 23% of the total ESBL respectively. Isolates from 12 animals presented the combination of both, ESBL and AmpC genes. Finally, mammals and raptors shared the largest part of the detected ESBL types, and other minority gene variants, such as *bla*_CTX-M-3_ and *bla*_SHV-11_ or *bla*_SHV-167_ were only detected in mammals or raptors, respectively (Fig 3).

A high genetic diversity of CR encoding genes was observed in all *Enterobacterales*, with 40% (16/40) of the isolates harbouring 2 to 5 different resistance genes in the same isolate (Table 1). Furthermore, the carbapenemase-encoding gene OXA-48 was detected in *E*. *coli* and *P*. *mirabilis* isolated from European hedgehog and Barn owl, respectively (Table 1). Other carbapenemase-encoding genes tested were not found.

**Table 1:** Prevalence and antimicrobial resistance genotypes and phenotypes of beta-lactamase producing *Enterobacterales* detected in wildlife.

*Scientific name* (common name)	Total sample	AMR genes		Bacterial spp	Drug-resistance genes	Resistance phenotype to non-Beta-lactams
**Mammals (N = 104)**	**N**	**n**	**Prev**	** **	** **	
*Aetechinus algirus* (Algerian hedgehog)	3	2	67%	*Escherichia coli*	CMY-2	CIP, NAL, KAN, TMP
				*Klebsiella oxytoca*	CTX-M-3	GEN, ST, FF, CF, TET, COL, TMP
*Erinaceus europeus* (European hedgehog)	47	12	26%	*Escherichia coli*	CMY-2	nd
* *				*Escherichia coli*	CMY-2	KAN
* *				*Escherichia coli*	CMY-2	nd
* *				*Escherichia coli*	SHV-12	STR
* *				*Escherichia coli*	SHV-11,OXA-48	CIP, NAL, KAN, TET, SUL, TMP
* *				*Klebsiella pneumoniae*	CMY-1,CMY-2, SHV-1,TEM-1,CTX-M-15	CIP, GEN, ST, KAN, TET, SUL, TMP
* *				*Klebsiella pneumoniae*	SHV-11, TEM-1	CIP, NAL, GEN, STP, KAN, TET, SUL, TMP
* *				*Klebsiella pneumoniae*	SHV-28	CIP, NAL, GEN, STR, KAN, TET, COL, SUL, TMP
* *				*Klebsiella pneumoniae*	SHV-12	CIP, NAL, KAN, TET, SUL, TMP
* *				*Citrobacter freundii*	CMY-2, TEM-1	CIP, NAL, KAN, TET, TMP
* *				*Citrobacter freundii*	CMY-2, SHV-12	CIP, NAL, STR, KAN, TET, COL, SUL, TMP
* *				*Citrobacter freundii*	CMY-2	CIP, NAL
*Capreolus capreolus* (European roe deer)	2	1	na	*Enterobacter cloacae*	CMY-2	SUL
*Martes foina* (Beech marten)	2	1	na	*Citrobacter freundii*	CMY-2, SHV-12	CIP, NAL, GEN, TET, SUL, TMP
*Meles meles* (European badger)	1	1	na	*Escherichia coli*	SHV-12	CIP, NAL, CHL, SUL, TMP
*Mustela vison* (American mink)	13	1	8%	*Enterobacter cloacae*	CMY-2	SUL
**PREVALENCE IN MAMMALS**	**104**	**18**	**17.3%**			
**Raptors (n = 108)**	**N**	**n**	**Prev**			
*Accipiter gentilis* (northern goshawk)	13	3	23%	*Escherichia coli*	TEM-1	COL
				*Escherichia coli*	CMY-2	CIP, NAL
				*Proteus mirabilis*	CMY-1, CMY-2, SHV-28, TEM-1	CIP, NAL, GEN, STR, KAN, TET, SUL, TMP
*Accipiter nisus* (Eurasian sparrowhawk)	8	3	38%	*Escherichia coli*	CMY-1, SHV-1, TEM-1, CTX-M-15	CIP, NAL, KAN, TET, SUL, TMP
* *				*Escherichia coli*	TEM-1	CIP, TET, TMP
* *				*Serratia marcensis*	CMY-1, CTX-M-15	CIP, TET, COL, SUL, TMP
*Bubo bubo* (Eurasian eagle-owl)	1	1	na	*Escherichia coli*	CMY-1, SHV-167	nd
*Buteo buteo* (Common buzzard)	17	2	12%	*Escherichia coli*	SHV-12	ST, CHL, TET, SUL, TMP
* *				*Providencia alcalifaciens*	SHV-12	CIP, NAL,GEN,ST,KAN, FF,CHL, TET,SUL, TMP
*Strix aluco* (Tawny owl)	18	3	17%	*Klebsiella pneumoniae*	CMY-2, SHV-28	STR, SUL, TMP
				*Escherichia coli*	CMY-2, SHV-1	nd
				*Klebsiella pneumoniae*	SHV-12, CTX-M15	CIP
*Tyto alba* (Barn owl)	3	2	67%	*Escherichia coli*	CMY-2	CIP, NAL, STR, TET
				*Proteus mirabilis*	SHV-12,TEM-1, OXA-48	CIP, NAL, STR, KAN, CF, TET, COL, SUL,TMP
**Other birds (n = 83)**	**N**	**n**	**Prev**			
*Carduelis carduelis* (European goldfinch)	12	1	8%	*Citrobacter freundii*	CMY-2	CIP, NAL, GEN, STR, KAN, CHL, TET, SUL, TMP
*Carduelis choris* (European Greenfinch)	2	1	na	*Klebsiella pneumoniae*	CMY-1	CIP, NAL, KAN, FF, CHL, SUL
*Larus michahellis* (Yellow-legged gull)	7	1	14%	*Escherichia coli*	CTX-M-15	CIP, NAL, GEN, KAN, TET, SUL, TMP
*Serinus serinus* (European serin)	6	1	17%	*Klebsiella pneumoniae*	CMY-1, SHV-28	CIP, NAL, STR, KAN, TET, SUL, TMP
*Streptopelia decaocto* (Eur. collared dove)	1	1	na	*Citrobacter freundii*	CMY-2	FF, TMP
*Sylvia melanocephala* (Sardinian warbler)	6	2	33%	*Escherichia coli*	CMY-2	CIP, NAL
* *				*Providencia spp*	CTX-M-15, CMY-1	CIP, NAL, GEN, STR, KAN, CHL, TET, SUL, TMP
*Turdus merula* (Common blackbird)	8	1	13%	*Escherichia coli*	CMY-2	CIP, NAL, KAN, TMP
**PREVALENCE IN BIRDS**	**191**	**22**	**11.5%**			

CIP, Ciprofloxacin; NAL, Nalidixic acid; GEN, Gentamicin; STR, Streptomycin; KAN, Kanamycin; FF, Florfenicol; CHL, Chloramphenicol; TET, Tetracycline; COL, Colistin; SUL, Sulphametoxazole; TMP, Trimethoprim. nd, not detected.

Most of the ESBL/AmpC *Enterobacterales* isolated (92%), with the exception of *E*. *cloacae*, were multiresistant with a common resistance phenotype comprising β-lactams-quinolones-tetracycline-sulfamethoxazole/trimethoprim (Table 1). *K*. *pneumoniae* and *C*. *freundii* isolates both presented a multi-drug resistance profile including the resistance to aminoglycosides (Table 2). Moreover, 90% of the *K*. *pneumoniae* isolates were resistant to ciprofloxacin and sulphametoxazole, 70% to kanamycin, 55% to streptomycin, and 10% to florfenicol. Additionally, 83% of the tested *C*. *freundii* isolates exhibited resistance to trimethoprim and nalidixic acid and 67% to tetracycline (Table 2). Although none of the *mcr*- genes were detected in this study, the colistin resistant phenotype was observed in *Klebsiella* spp isolated from a European greenfinch and Algerian hedgehog, and in a *Providencia* spp isolated from a common buzzard.

**Table 2:** Minimal inhibitory concentration of *E*. *coli*, *K*. *pneumoniae* and *C*. *freundii* isolates of wildlife origin. Dilution ranges for each antimicrobial are those contained within the white area. Vertical lines indicate epidemiological cut off values (ECOFF) or clinical breakpoints in those cases where ECOFF values have not been described.

***E*. *coli* (n = 18)**															
MIC [μg/mL]:	0.12	0.25	0.5	1	>2	4	8	16	>16	32	64	>128	256	>512	R (%)
Ampicilin											3	15			100
Cefotaxime	1	1	1	2	12	1									89
Ceftazidime				2		1	1	7	7						100
Ciprofloxacin	6	2	2	8											67
Nalidixic acid					2	3	4					9			50
Gentamicin			9	8						1					5.5
Streptomycin						7	3	5		2	1				17
Kanamycin							12	2	4						33
Florfenicol						4	12	2							0
Chloramphenicol					2	8	5	1		2					11
Tetracycline				6	6					1	2	3			33
Colistin			17			1									5.5
Sulphametoxazole							2	9		1	1			5	28
Trimethoprim	1	7	2							8					44
***K*. *pneumoniae* (n = 9)**															
MIC [μg/mL]:	0.12	0.25	0.5	1	>2	4	8	16	>16	32	64	>128	256	>512	R (%)
Ampicilin^a^											1	8			100
Cefotaxime					9										100
Ceftazidime							3		6						100
Ciprofloxacin		1		8											100
Nalidixic acid^a^						1			2	1		5			88
Gentamicin		1	5						3						33
Streptomycin						2			2		2	3			ND
Kanamycin^a^							2		7						78
Florfenicol						1	7			1					ND
Chloramphenicol^a^					1	5	1		1	1					22
Tetracycline				1	1		2			1		4			56
Colistin			8			1									11
Sulphametoxazole^b^									1					8	89
Trimethoprim^b^		1			2				6						67
***C*. *freundii* (n = 6)**															
MIC [μg/mL]:	0.12	0.25	0.5	1	>2	4	8	16	>16	32	64	>128	256	>512	R (%)
Ampicilin^a^											1	5			100
Cefotaxime	1				5										83
Ceftazidime				1			1		4						83
Ciprofloxacin^b^	1			5											0
Nalidixic acid^a^						1						5			83
Gentamicin		1	1			2			2						33
Streptomycin					1	1			2	1			1		ND
Kanamycin^b^							3		3						50
Florfenicol						3	2		1						ND
Chloramphenicol^a^					1	2	1			1	1				33
Tetracycline				2					1	2	1				67
Colistin^a^			4		1	1									33
Sulphametoxazole^b^							2		1					3	50
Trimethoprim^b^		1							5						83

EUCAST (ECOFF values WT): AMP ≤8, CTX ≤0.25, CAZ ≤0.5 (≤1 *C*.*freundii*), CIP ≤0.064 (≤0.125 *K*.*pneumoniae*), NAL ≤16, GEN ≤2, STR≤ 16, KAN ≤8, FFL ≤16, CHL ≤16, TET ≤8, COL ≤2, SMX ≤64, TMP ≤2 (≤8 *K*. *pneumoniae* and *C*. *freundii*).

^a^BSAC and

^b^CLSI clinical break points: CIP ≤1 Enterobacteriaceae, SMX Susceptible ≤256 *K*. *pneumoniae* and *C*. *freundii*; TMP ≤8 *K*. *pneumoniae* and *C*. *freundii*. ND, not determined due to lack of ECOFF or clinical breakpoint values available. CLSI: Performance Standards for Antimicrobial Susceptibility Testing. 27th ed. CLSI supplement M100. Wayne, PA: Clinical and Laboratory Standards Institute; 2017.

## Discussion

This study identifies for the first time a high percentage of wild mammals and wild birds as carriers of potential nosocomial *Enterobacterales* harbouring diverse ESBL, CMY and OXA-48 genes. Moreover, most of the isolates principally *K*. *pneumoniae* and *C*. *freundii*, presented a high prevalence of resistance also to fluoroquinolones.

In general, *E*. *coli* is the most reported ESBL/pAmpC-producing enterobacteria worldwide, with increasing frequency from animals, food, environmental sources and humans. In recent years, CR- *E*. *coli* transmission has been reported in different hosts, demonstrating a close human-animal ESBL/pAmpC gene similarity between livestock (broilers and pigs) and personnel working at the farms [13]. Additionally, similar CR genes have been reported between isolates from the community and those from human clinical settings, sewage water and wild birds [13].

Although ESBL transmission has been studied extensively in *Enterobacterales* from humans and livestock, data on antimicrobial resistance in the environment is still limited [2]. Moreover, most of the studies related to ESBL-carrying bacteria in wildlife are focused on the wild bird population and mainly restricted to *E*. *coli* species [32]. Several studies conducted in *E*. *coli* from avian species have identified *bla*_CTX-M-1_, *bla*_CTX-M-14,_ *bla*_CTX-M-15_ and *bla*_SHV-12_ as the predominant ESBL types circulating in Spain [15, 33–36], Portugal [37], Tunisia [20], The Netherlands [38], Poland [39] and the Czech Republic [40]. Contrarily, in the present study, *bla*_CTX-M-1_ and *bla*_CTX-M-14_ were not detected in our avian species, but *bla*_SHV-12_ and *bla*_CTX-M-15_ were the most frequent ESBL types identified not only in *E*. *coli* but also in *K*. *pneumoniae* and *C*. *freundii* isolates. *K*. *pneumoniae* has been described in low prevalence (average 1.5%) in wild gulls from different European countries [41–43], including wild migratory birds from Spain, which exhibited *bla*_CTX-M-15_ ESBL-producing *K*. *pneumoniae* [36]. Interestingly, both *bla*_CTX-M-15_ and *bla*_SHV-12_ are also currently the most predominant genes in human clinical specimens from community and health care-associated infections in Spain [44,45]. Thus, the human community could potentially be a source of ESBL environmental contamination, through water contaminated with human sewage from urban areas and hospital settings.

In this study, *bla*_CMY-1_ group was principally detected in *E*. *coli*, *K*. *pneumoniae*, *Proteus* and *Providencia* spp from avian wildlife, like hawks, owls and small forest birds. Although this is an unusual variant in Spain, the presence in the present study might be explained by those species feeding habits. Raptors are predators occupying the top of the food chain; therefore, they can acquire AMR from a wide variety of preys (mammals, birds, reptiles or scavenging livestock). Moreover, some of these raptors are migratory species, being exposed to different environmental habitats in their migratory movements. In consequence, the role of migratory raptors as disseminators of these AMR traits is a serious concern to be further investigated.

Regarding *bla*_CMY-2,_ it is the most common CMY type reported worldwide [46]. In this study, *bla*_CMY-2_ group was highly detected in *E*. *coli* and *K*. *pneumoniae* from hedgehogs and wild birds. Plasmid mediated genes can spread easily to other organisms. *C*. *freundii*, *Enterobacter* and *Serratia* spp in this study presented genes of the CMY-2 family. Since these types of AmpC genes are chromosomally encoded in some of these bacteria species, we cannot conclude the plasmidic nature of such enzymes. However, for epidemiological studies, it is important to report this type of resistance since these *Enterobacterales* can be involved in severe nosocomial infections and they all presented a MDR profile, except for *E*. *cloacae*.

Surprisingly, European hedgehogs represented an important reservoir of ESBL/AmpC-producing *E*. *coli* and other *Enterobacterales*, especially for *bla*_CMY-2_ (67%) and *bla*_SHV-12_ (25%) in this study. Our results are in agreement with previous studies conducted in Spain reporting low to moderate (1.3%-10%) prevalence of *bla*_CMY-2_ and *bla*_SHV-12_ *E*. *coli* variants in hedgehogs, deer and minks [22,47]. It is important to highlight that hedgehogs are in close contact with humans (home range including gardens), but also with livestock in the countryside, which could explain their acquisition of these AMR types.

Plasmid-mediated colistin resistance by *mcr*-1 has been reported worldwide in *Enterobacterales* isolated from humans, livestock, companion animals, food and wildlife [48]. Colistin has been used in veterinary medicine during the last decades for the treatment of gastrointestinal infections in livestock, principally in pigs and poultry [49]. Consequently, livestock is considered the main reservoir of *mcr*-1 selection and dissemination worldwide. Recent works disclosed the relationship among *mcr*-1-harbouring *E*. *coli* isolates recovered from the environment, pig production and human clinical isolates, demonstrating the rapidly evolving epidemiology of plasmid-mediated colistin-resistant *E*. *coli* strains worldwide and the importance of the One Health approach [50,51]. In our study, some *Klebsiella* and *Providencia* spp isolates were phenotypically resistant to colistin, but no *mcr*-associated genes were detected in any of the examined isolates.

Information about carbapenem-resistant *Enterobacterales* is very scarce in wildlife and has only been reported in avian species [36,52]. In this study, we report the presence of *bla*_OXA-48_ in *E*. *coli* and *P*. *mirabilis* isolates from a European hedgehog and a Barn owl, respectively. The presence of *bla*_OXA-48_ in wild mammals and birds in Catalonia is highly indicative of the wide environmental pollution of this variant, commonly reported in hospitals in Spain [53].

To our knowledge, there are no reports in wildlife, especially in wild mammals, describing the presence of ESBL genes in such a variety of *Enterobacterales*, like *Klebsiella* spp, *Citrobacter* spp, *Serratia* spp, or *Enterobacter* spp in Spain. *C*. *freundii*, is considered an opportunistic pathogen, associated with nosocomial infections, especially in patients who have been hospitalized for a prolonged period of time. In the last years, this bacterium has been classified as an emerging health problem associated to urinary tract infections commonly diagnosed in healthcare settings [54]. *E*. *cloacae* has been reported as important opportunistic and multi-resistant pathogen involved in outbreaks of hospital-acquired infections worldwide [55,56], including Spain [57]. ESBL- *S*. *marcescens* has also been classified as one of the top ten priority pathogens causing infections in intensive care units [58].

The high prevalence of CR *Enterobacterales* encountered in this study is really concerning, since wildlife is not directly exposed to any antimicrobial agent. Therefore, faecal contamination of water or soil with MDR bacteria and/or antimicrobial residues can lead to a selection pressure. Wastewaters from urban areas and hospitals have been identified as one of the major sources of AMR environmental contamination [2]. High prevalence of *bla*_SHV-12_ but also *bla*_TEM-1_ and *bla*_CTX-M-1_ alleles have been reported in aquatic environments (urban waters, natural or artificial water reservoirs, seawater or drinking water) in several countries worldwide, likely due to their relatively easy transmission to surface water through waste water treatment plant discharges [2,59]. In our study, wildlife in close contact with urban and farming areas of Catalonia carried a large variety of zoonotic/nosocomial bacteria genetically resistant to β-lactams-quinolones-tetracycline-sulfamethoxazole/trimethoprim-aminoglycosides with similar resistant genes to those found in livestock and clinical settings. However, further studies are needed to assess clonal relatedness among different cephalosporin and carbapenem resistant enterobacteria at the human-animal-environment interface.

## Conclusions

This study describes for the first time a high prevalence of *Enterobacterales* harbouring a large variety of ESBL in addition to carbapenem resistant OXA-48 genes in wild mammals, remarkably in hedgehogs, and wild birds in Catalonia (northeast Spain). AmpC CMY-2 group and the ESBL genes *bla*_SHV-12_ and *bla*_CTX-M-15_ were the most frequent types identified in *E*. *coli*, *K*. *pneumoniae* and *C*. *freundii* isolates. These results support the concept that wildlife is a good sentinel of AMR environmental contamination and underline the importance of the One Health approach since wildlife can contribute indirectly to the dissemination of resistance genes into other natural environments.

## Supporting information

S1 Table Oligonucleotides used for the detection of ESBL/AmpC and colistin-resistance genes in this study. F, sense primer; R, antisense primer; bp, base pairs.
(DOCX)
