# Supplementary material for: High prevalence and diversity of extended-spectrum β-lactamase and emergence of OXA-48 producing Enterobacterales in wildlife in Catalonia
Source: PLoS One. 2019 Aug 5;14(8):e0210686. doi: 10.1371/journal.pone.0210686 (PMC6681944; doi:10.1371/journal.pone.0210686)
Supplement: S1 Table — F, sense primer; R, antisense primer; bp, base pairs. (DOCX) [file pone.0210686.s001.docx]

**S1Table**. **Oligonucleotides used for the detection of ESBL/AmpC and colistin-resistance genes in this study**.

| Genes |  | Primer sequence (5’-3) | Product size (bp) | References |
| --- | --- | --- | --- | --- |
| bla_TEM_ | F | GCGGAACCCCTATTTG | 963 | Olesen *et al.,* 2004 |
|  | R | TCTAAAGTATATATGAGTAAACTTGGTCTGAC |  |  |
| bla_CTX-M_ | F | ATGTGCAGYACCAGTAARGTKATGGC | 593 | Miro *et al.,* 2002 |
|  | R | TGGGTRAARTARGTSACCAGAAYCAGCGG |  |  |
| bla_SHV_ | F | TTATCTCCCTGTTAGCCACC | 797 | Arlet *et al.,* 1997 |
|  | R | GATTTGCTGATTTCGCTCGG |  |  |
| bla_CMY-1_ | F | ATGCAACAACGACAATCC | 1085 | Kim *et al.,* 1998 |
|  | R | TTGGCCAGCATGACGATG |  |  |
| bla_CMY-2_ | F | GCACTTAGCCACCTATACGGCAG | 758 | Hasman *et al.,* 2005 |
|  | R | GCTTTTCAAGAATGCGCCAGG |  |  |
| bla_OXA-48_ | F | GCGTGGTTAAGGATGAACAG | 438 | Poirel *et al.,* 2011 |
|  | R | CATCAAGTTCAACCCAACCG |  |  |
| bla_VIM_ | F | GATGGTGTTTGGTCGCATA | 390 | Poirel *et al.,* 2011 |
|  | R | CGAATGCGCAGCACCAG |  |  |
| bla_IMP_ | F | GGAATAGAGTGGCTTAAYTCTC | 232 | Poirel *et al.,* 2011 |
|  | R | CCAAACYACTASGTTATCT |  |  |
| bla_NDM_ | F | GGTTTGGCGATCTGGTTTTC | 621 | Poirel *et al.,* 2011 |
|  | R | CGGAATGGCTCATCACGATC |  |  |
| bla_KPC_ | F | CGTCTAGTTCTGCTGTCTTG | Variable | Poirel *et al.,* 2011 |
|  | R | CTTGTCATCCTTGTTAGGCG |  |  |
| *mcr-*1 | F | CGGTCAGTCCGTTTGTTC | 309 | Rebelo *et al.,* 2018 |
|  | R | CTTGGTCGGTCTGTAGGG |  |  |
| *mcr-*2 | F | TGTTGCTTGTGCCGATTGGA | 567 | Rebelo *et al.,* 2018 |
|  | R | AGATGGTATTGTTGGTTGCTG |  |  |
| *mcr-*3 | F | TTGGCACTGTATTTTGCATTT | 542 | Rebelo *et al.,* 2018 |
|  | R | TTAACGAAATTGGCTGGAACA |  |  |
| *mcr-*4 | F | ATTGGGATAGTCGCCTTTTT | 487 | Rebelo *et al.,* 2018 |
|  | R | TTACAGCCAGAATCATTATCA |  |  |
| *mcr-*5 | F | ATGCGGTTGTCTGCATTTATC | 1644 | Rebelo *et al.,* 2018 |
|  | R | TCATTGTGGTTGTCCTTTTCTG |  |  |

F, sense primer; R, antisense primer; bp, base pairs
